# Vascular complications of diabetes: A narrative review

**DOI:** 10.1097/MD.0000000000035285

**Published:** 2023-10-06

**Authors:** Yongxia Lu, Wei Wang, Jingyu Liu, Min Xie, Qiang Liu, Sufang Li

**Affiliations:** a Department of Endocrinology and Metabolism, Chengdu Seventh People’s Hospital, Chengdu, China; b Department of Cardiovascular Medicine, Chengdu Seventh People’s Hospital, Chengdu, China.

**Keywords:** complications, diabetes mellitus, macrovascular, microvascular, risk factors

## Abstract

Diabetes mellitus is a complex chronic metabolic disease characterized by hyperglycemia and various complications. According to the different pathophysiological mechanisms, these complications can be classified as microvascular or macrovascular complications, which have long-term negative effects on vital organs such as the eyes, kidneys, heart, and brain, and lead to increased patient mortality. Diabetes mellitus is a major global health issue, and its incidence and prevalence have increased significantly in recent years. Moreover, the incidence is expected to continue to rise as more people adopt a Western lifestyle and diet. Thus, it is essential to understand the epidemiology, pathogenesis, risk factors, and treatment of vascular complications to aid patients in managing the disease effectively. This paper provides a comprehensive review of the literature to clarify the above content. Furthermore, this paper also delves into the correlation between novel risk factors, such as long noncoding RNAs, gut microbiota, and nonalcoholic fatty liver disease, with diabetic vascular complications.

## 1. Introduction

**Figure 1.**
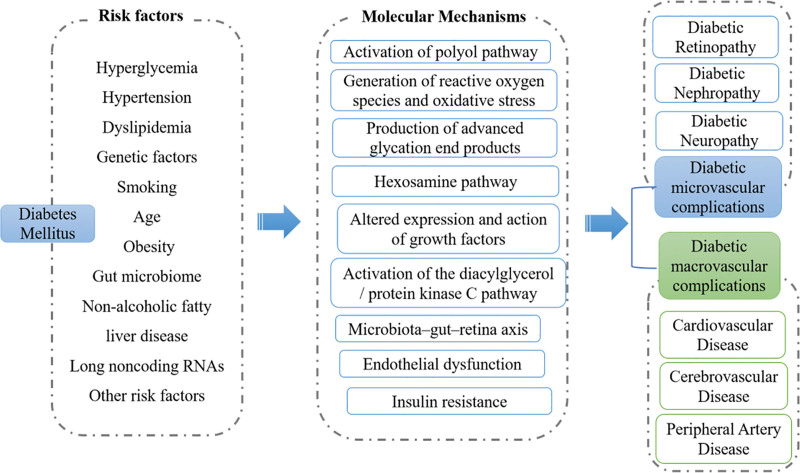
The important molecular mechanisms of diabetic vascular complications. Numerous risk factors contribute to the development of diabetic vascular complications, such as hyperglycemia, hypertension, dyslipidemia, genetic factors, and smoking. Moreover, recent studies have revealed associations between the gut microbiome, nonalcoholic fatty liver disease, long noncoding RNAs, and an elevated incidence of diabetic vascular complications. The mechanisms underlying the influence of the aforementioned risk factors on the occurrence of diabetic vascular complications are complex, involving multiple pathways such as the activation of the polyol pathway, the generation of reactive oxygen species and oxidative stress, as well as the production of advanced glycation end products.

Diabetes mellitus (DM) is a well-known metabolic disease characterized by organ dysfunction that arises directly or indirectly from the effects of chronic hyperglycemia. It affects a significant proportion of the global population. According to the International Diabetes Federation, the worldwide prevalence of diabetes was estimated to be 9.3% in 2019, with 463 million people living with the disease. Furthermore, the prevalence of diabetes is projected to increase to 10.2% (578 million) by 2030 and 10.9% (700 million) by 2045.^[1]^ Moreover, DM is a leading cause of mortality and disability worldwide, with vascular complications being a significant contributor, accounting for 26.8%.^[2]^ Diabetic vascular complications not only threaten the quality of life and longevity of patients with diabetes but also impose a significant economic burden on individuals and healthcare systems worldwide. Therefore, healthcare providers must have a comprehensive understanding of vascular complications to identify new strategies for early intervention or prevention. This review examines the epidemiology, risk factors (Table 1), and mechanisms that contribute to the development of various vascular complications in patients with diabetes (Fig. 1). Additionally, we provide a brief overview of prevention and screening strategies for representative complications.

**Table 1:** Risk factors of diabetic vascular complications.

Disease	Risk factors
Microvascular complications	
Diabetic retinopathy	Duration of diabetes, Hyperglycemia, Hypertension, Genetic factors, Nephropathy, Smoking, Dyslipidemia, BMI, Gut microbiome, NAFLD, lncRNAs
Diabetic nephropathy	Hyperglycemia, Hypertension, Genetic factors, Smoking, Dyslipidemia, Gut microbiome, Obesity, Race, Gender, Age, NAFLD, lncRNAs
Diabetic neuropathy	Duration of diabetes, Hyperglycemia, Hypertension, Genetic factors, Smoking, Age, higher levels of total and low-density lipoprotein cholesterol and triglycerides, higher body-mass index, higher von Willebrand factor levels, UAE rate, NAFLD
Macrovascular complications	
Cardiovascular disease	Hyperglycemia, Hypertension, Dyslipidemia Obesity, Diabetic nephropathy, Genetic factors, Age, NAFLD
Cerebrovascular disease	Hyperglycemia, Hypertension, Genetic factors, Smoking, Microbiota, Obesity, High-density lipoprotein cholesterol, history of vascular disease, Heart failure, Atrial fibrillation
Peripheral artery disease	Hyperglycemia, Obesity, Gender, Age, elevated serum lipoprotein levels, Insulin resistance, Elevated serum fibrinogen levels, Microalbuminuria, Increased levels of intercellular adhesion molecule

BMI = body mass index, LncRNAs = long noncoding RNAs, NAFLD = nonalcoholic fatty liver disease, UAE = urine albumin excretion.

## 2. Microvascular complications

With the progress of DM, patients are more likely to develop various vascular complications, which are classified as microvascular or macrovascular complications depending on the underlying pathophysiology. Microvascular diseases contribute to pathologic and functional changes in many tissues, including eye, heart, kidney, skin, and neuronal tissues. These changes are traditionally known as diabetic retinopathy (DR), nephropathy, peripheral neuropathy, and autonomic neuropathy, respectively, based on the tissues affected.

Microvascular complications are an important cause of significant increases in morbidity and considerable impairment of quality of life in patients with diabetes. In the past decades, several classical hypotheses have then been proposed to explain the process of developing microvascular complications, including activation of polyol pathway, generation of reactive oxygen species (ROS) and oxidative stress, production of advanced glycation end products, initiation of flux through the hexosamine pathway, altered expression and action of growth factors, and activation of the diacylglycerol/protein kinase C pathway (Fig. 1).^[3]^ In addition, some novel mechanisms of diabetic microvascular complications have been proposed recently. For instance, the potential mechanisms of long noncoding RNAs in the development of diabetic nephropathy (DN) and DR, which relates to the roles of long noncoding RNAs in mesangial cells proliferation and fibrosis, inflammatory processes, extracellular matrix accumulation in the glomeruli and tubular injury, as well as the role of abnormal neovascularization and neuronal dysfunction.^[4,5]^ nonalcoholic fatty liver disease (NAFLD) was viewed both as a driving force and a consequence of the DM.^[6]^ Meanwhile, accumulating evidence suggests that the presence of microvascular complications (including DR, DN, and peripheral neuropathy) was inversely associated with NAFLD among hospitalized patients with type 2 diabetes.^[7–9]^ Thus, NAFLD should be used to stratify the risk of diabetic complications and incorporated into disease management programs for patients with diabetes. Recently, Giovanni Targher et al discuss the putative underlying mechanisms by which NAFLD contributes to vascular diseases. It is worth noting that they also proposed that altered gut microbiota in patients with diabetes lead to intestinal barrier dysfunction, which might in turn increases the risk of vascular complications associated with NAFLD through various potential pathways, factors, and processes.^[9]^ However, further studies in patients with diabetes are required to better characterize the relationship.

### 2.1. Diabetic retinopathy

DR is a common microvascular complication of diabetes, which is usually subdivided into the earlier stage of non-proliferative DR and the advanced stage of proliferative DR. Moreover, macular edema can occur at any stage of DR and pose a threat to vision. The clinical findings of non-proliferative DR include retinal microaneurysms, cotton wool spots, hemorrhages, and exudates, which the patient may not be aware of. While proliferative DR is typically characterized by the formation of pathologic preretinal neovascularization that is very fragile and can thus rupture and bleed profusely.^[10]^

A pooled analysis of 35 studies conducted globally between 1980 and 2008 revealed an overall prevalence of 34.6% (95% CI, 34.5–34.8) for any form of DR, which includes non-proliferative DR, proliferative DR, diabetic macular edema, or any combination of these conditions. The prevalence of proliferative DR, diabetic macular edema, and vision-threatening DR among people with diabetes was 6.96% (6.87–7.04), 6.81% (6.74–6.89), and 10.2% (10.1–10.3), respectively.^[11]^ In contrast to the global prevalence, DR affects a slightly lower proportion of the diabetic population in the United States, with a prevalence of 28.5% and a proportion of 4.4% with threatened loss of vision.^[12]^ DR is the primary cause of visual loss in the elderly, especially in middle- and high-income countries, responsible for an estimated 10,000 new cases of blindness annually.^[13,14]^ Moreover, the crude prevalence of visual impairment and blindness caused due to DR increased substantially between 1990 and 2015, according to the report of the Vision Loss Expert Group of the Global Burden of Disease Study,^[15]^ which may be related to the increasing prevalence of type 2 diabetes in low- and middle-income countries. Conversely, the situation in the United States is relatively positive, with the percentage of diabetic patients with visual impairment declining from 26% in 1997 to 19% in 2011, while the overall rate of visual impairment among the general population remained stable at 9.3%.^[16]^ This trend may be attributed to improved treatment and care measures in the United States.

The occurrence and development of DR strongly correlate with a longer duration of diabetes and chronic hyperglycemia, and hypertension. For instance, a recent study found that diabetes duration is significantly associated with DR with OR = 1.098 (95% CI, 1.068–1.129) based on regression analysis.^[17]^ Several studies have confirmed the association between greater hyperglycemia and DR.^[18–20]^ Stratton IM et al found that each 1% reduction in updated mean HbA1c was associated with a 37% reduction in risk for microvascular complications (mostly retinopathy) (33%–41%, *P* < .0001).^[21]^ Similarly, ample evidence confirms the relationship between hypertension and DR.^[20,22,23]^ Additionally, CACNB2 was identified as a susceptibility gene for DR, which is abundantly expressed in retinal cells and encodes the β2 subunit of the L-type calcium channel, regulating the expression of VEGF.^[24]^ In addition to the risk factors described above, other factors associated with DR include nephropathy, smoking, dyslipidemia, higher body mass index and gut microbiome (Table 1).^[20,24–26]^

Although a significant amount of data has been collected and analyzed on diabetes, the development and severity of DR are not yet fully explained by known risk factors, and the molecular mechanism behind this disease requires further understanding. Presently, oxidative stress, inflammation, neovascularization, neurodegeneration, and neurovascular unit are known to be mechanisms of DR occurrence and development. Elevated intracellular glucose in patients with diabetes leads to oxidative stress, which causes a rise in intracellular ROS, through trigger the polyol pathway and hexokinase pathway, which metabolizes glucose. It can lead to pyroptosis, apoptosis, and autophagy, promoting inflammation, vascular degeneration, neurodegeneration, and neovascularization.^[27–30]^

Alongside these mechanisms, accumulating studies emphasized a reliable connection between gut microbiome and DR which is considered to be a “microbiota–gut–retina axis.”^[26,31–35]^ The gut microbiota promotes the occurrence and development of DR through mechanisms such as inflammation, barrier dysfunction, vascular permeability, and metabolites.^[26]^ Compared with healthy individuals, the intestinal microecology of patients with diabetes without retinopathy and DR patients is dysregulated.^[34]^ Dysbiosis of intestinal flora increases intestinal permeability, leading to endotoxemia, which allows metabolites to freely enter the bloodstream, leading to chronic inflammation and immune system imbalance. Subsequently, inflammatory signals are cascade amplified to promote the occurrence of DR.^[26]^ However, direct evidence that gut microbiota cause DR remains weak, and further cellular and molecular studies, as well as well-controlled human studies, are needed for verification.

### 2.2. Diabetic nephropathy

DN, also known as diabetic kidney disease, is a common and severe complication of diabetes that leads to chronic kidney disease. Approximately 1 in 5 patients with diabetes are affected. The pathology of DN is characterized by pathological levels of urine albumin excretion (UAE), diabetic glomerular lesions, and loss of glomerular filtration rate (GFR) in patients with diabetes.^[36]^ The classic definition of DN involves the presence of proteinuria >0.5 g/24 hours, while it is now defined by increased urinary albumin excretio in the absence of other renal diseases.^[37]^ Based on the values of UAE, DN is divided into microalbuminuria (UAE >20 μg/min and ≤199 μg/min) and macroalbuminuria (UAE ≥200 μg/min) stages.

The incidence of DN varies by ethnicity and level of economic development, generally ranging from 20% to 30%. The overall pooled prevalence of DN among patients with type 2 diabetes in China was 21.8% (95% CI: 18.5%–25.4%), and the prevalence of DN was also high at 26.1% in the population of urban type 2 diabetes patients in South India.^[38]^ However, Khalid et al found a lower overall prevalence of DN (10.8%) among patients with type 2 diabetes aged ≥25 years from the Saudi National Diabetes Registry compared to the Chinese and Indian populations.^[39]^ Unfortunately, the prevalence has not declined over the past few decades in developing countries,^[40]^ while DN in parts of Western and Northern Europe, as well as the United States, has decreased.^[41,42]^ Therefore, it can be concluded that DN is more severe in developing countries than in developed ones.

The risk factors associated with the development and progression of DN have been extensively investigated, similar to DR. Chronic hyperglycemia, high blood pressure, and genetic predisposition are the primary risk factors for DN. Additionally, other contributing factors include obesity, smoking, race, dyslipidemia, male gender, and age.^[43–45]^ A study has shown that patients with type 1 diabetes who smoke and have HbA(1c) >8% are at an increased risk of developing microalbuminuria.^[46]^ African Americans, Mexican Americans, and Pima Indians are at higher risk for DN due to their racial classification.^[37]^ Genetic factors also play a significant role in the onset of DN. A large meta-analysis found that 24 genetic variants in 16 genes were associated with DN.^[47]^ Furthermore, DR presence predicts faster DN progression.^[48]^

DN is a complex multifactorial disease. The pathogenesis of DN involves various pathways and mediators, such as cellular senescence, which plays an important role in the onset and development of DN. The mechanisms of cellular senescence involve several factors, including telomere attrition, DNA damage, epigenetic changes, mitochondrial dysfunction, Klotho loss, activation of the Wnt/β-catenin signaling pathway, persistent inflammation, and uremic toxin accumulation.^[49]^ Immune inflammation, epithelial-mesenchymal transition, apoptosis and mitochondrial damage, epigenetics, and podocyte-endothelial communication are also crucial pathways for the development and progression of DN.^[50]^ Furthermore, there is significant overlap and interaction among these pathways. For example, Nicotinamide adenine phosphate dehydrogenase oxidase increases TGF-β, and conversely, TGF-β increases ROS through activation of nicotinamide adenine phosphate dehydrogenase oxidase. As such, the exact pathogenic mechanism and molecular incidence of DN remain unclear and the contribution of each pathway to the onset of DN is not yet confirmed.^[51]^ Therefore, further studies are necessary to identify the mechanisms of DN development for effective prevention and treatment against DN.

### 2.3. Diabetic neuropathy

Diabetic neuropathy is a clinical syndrome characterized by pain due to somatosensory nervous system lesions. Based on the affected neurons in the brain, diabetic neuropathy can be classified into peripheral, autonomic, proximal, and focal types, each presenting with various symptoms such as numbness, stomach issues, and cardiac problems.^[52]^ Peripheral diabetic sensory neuropathy is the most common presentation of microvascular complication and affects up to 50% of individuals with long-standing diabetes.^[53,54]^ According to a study conducted on the Dutch polyneuropathy population, diabetes accounted for 32%, and the frequency of diabetic neuropathy increased with age.^[55]^ In addition, the incidence of diabetic neuropathy is directly related to the duration of diabetes. Another study found that the prevalence of diabetic neuropathy among newly diagnosed patients with non-insulin-dependent DM was 8.3% at baseline and increased to 41.9% after 10 years.^[56]^ Like DR and DN, HbA(1c) levels are also risk factors for diabetic neuropathy. Furthermore, metabolic risk factors such as higher levels of total and low-density lipoprotein cholesterol and triglycerides, higher body-mass index, higher von Willebrand factor levels, UAE rate, hypertension, and smoking have all been closely associated with the cumulative incidence of neuropathy.^[57,58]^ Genetic studies have identified several genes linked to diabetic neuropathy; however, more verification in larger populations, especially with cohort studies, is needed.^[59]^

Several mechanisms for the development of diabetic neuropathy have emerged from recent studies. Rashmi Pathak et al proposed that the polyol pathway, advanced glycation end products pathway, inflammation, hexosamine pathway, protein kinase C pathway, Poly (ADP-ribose) polymerase pathway, and oxidative stress may be involved in the pathological processes leading to diabetic neuropathy.^[60]^ Hyperglycemia activates the polyol pathway. Non-enzymatic glycation and oxidative damage may act together or independently, directly affecting nerve tissue or nourishing vascular tissue, causing diabetic neuropathy.^[61,62]^

## 3. Macrovascular complications

Macrovascular diseases affect large blood vessels such as arteries and veins, including cardiovascular disease (CVD), cerebrovascular disease (CeVD), and peripheral arterial disease (PAD) lesions.^[63]^ These complications occur in approximately 20% to 30% of patients with diabetes, contributing significantly to the morbidity and mortality rate of type 2 diabetes.^[64–66]^

In recent years, there has been a significant decline in the risk of CVD among patients with diabetes due to better recognition of the negative impact of diabetes and the availability of novel pharmacological reagents. For instance, modifiable CVD risks among diabetic patients decreased from 37.7% in 2003 to 19.1% in 2008 in the Swedish population, and a similar trend was observed in other 2 studies.^[67–69]^ From 1999 to 2000, the estimated 10-year risk for CVD among patients with diabetes in the UK prospective diabetes study algorithms was 21.1%; however, it decreased to 16.4% in 2007 to 2008.^[69]^ Similar to CVD, the prevalence of diabetes combined with CeVD also showed a downward trend in enrolled patients with diabetes, while the prevalence of PAD in patients with diabetes is increasing.^[66]^

The key pathological mechanism of macrovascular complications is assumed to be an injury to the vascular endothelium. Furthermore, impaired platelet function may lead to an increased risk of thrombosis and atherosclerosis progression, which is related to the development and progression of diabetic macrovascular complications.^[70]^ Hyperglycemia, obesity, insulin resistance, and other factors increase the generation of ROS through different pathogenetic pathways, which lower the activity of NO by their NO-inactivating effect. NO is a key molecule in maintaining endothelial cell function; thus, insulin resistance and obesity lead to endothelial dysfunction and subsequent atherosclerotic changes due to reduced NO activity.^[71]^

Furthermore, several studies have found that the increased risk of macrovascular complications, especially coronary artery disease (CAD), is also closely related to NAFLD.^[72–74]^ Mechanisms explaining this relationship include the regulation of insulin resistance by hepatic lipid accumulation, the release of pro-inflammatory, pro-oxidative, and pro-coagulant factors and profibrotic mediators in the bloodstream, oxidative stress and hypertension, which can promote myocardial remodeling and dysfunction, thereby inducing the occurrence of various cardiac complications.^[73,75]^

### 3.1. Cardiovascular disease

CVD is the primary cause of death among type 2 diabetes patients, accounting for over 70% of fatalities.^[76]^ For decades, it has been established that diabetes increases the risk of developing CVD. After controlling for traditional CVD risk factors such as age, obesity, tobacco smoking, dyslipidemia, and hypertension, individuals with diabetes have a 4-fold greater chance of experiencing a CVD episode than those without diabetes.^[77,78]^

CAD, also known as ischemic heart disease, coronary heart disease (CHD), atherosclerotic heart disease, and atherosclerotic CVD, is a form of CVD that manifests as stable angina pectoris, unstable angina pectoris, myocardial infarction (also referred to as a heart attack), and sudden cardiac death.^[79]^ Globally, CHD affects approximately 32.2% of all individuals with type 2 diabetes.^[80]^ Additionally, DM is strongly associated with an elevated risk of CAD, such that patients with diabetes have a considerably higher 7-year risk of myocardial infarction than those without diabetes, regardless of the presence of prior myocardial infarction history.^[81,82]^ In contrast, a meta-analysis consisting of 13 studies with a total of 45,108 patients suggested that patients with diabetes without prior myocardial infarction had a 43% lower risk of developing total CHD events compared with those without diabetes but with a history of myocardial infarction.^[83]^ Although the difference in risks between the 2 studies may be related to the different thresholds defined for patients with diabetes,^[16]^ there is no doubt that diabetes increases the risk of future myocardial infarction, particularly for women.^[84]^ Moreover, diabetes can intensify the adverse consequences of myocardial infarction. In the TIMI-50 trial that evaluated the efficacy of vorapaxar in patients with and without diabetes, patients with diabetes had a more frequent incidence of cardiovascular death, myocardial infarction, or stroke than those without diabetes.^[85]^ In addition, recent genome-wide studies have unveiled genetic variation and epigenetic alterations as risk factors for CAD complications in patients with diabetes.^[86]^ Jeanette Erdmann et al summarized 163 CAD risk loci based on 10 years of genome-wide association studies.^[65]^ These loci may be useful for improved prediction of CAD risk and for encouraging preventive measures. The other risk factors for CAD are show in Table 1.^[87]^

### 3.2. Cerebrovascular disease

The CeVD affects 20% to 40% of individuals with type 2 diabetes and is a leading cause of mortality and severe morbidity worldwide.^[86]^ CeVD encompasses a range of medical conditions affecting the cerebral vessels and cerebral circulation and is characterized by stroke as a significant manifestation. It can be classified into ischemic or hemorrhagic CeVD. In 2019, stroke caused 6.6 million deaths globally, of which half were due to ischemic stroke, 44% (2.9 million) from intracerebral hemorrhage, and 6% (0.4 million) from subarachnoid hemorrhage.^[88]^ Furthermore, though the overall number of stroke-related deaths has been rising, there has been a 14.7% decrease in the age-standardized death rate for the 9-year period from 2010 to 2019.^[88,89]^

DM is associated with an elevated risk of stroke. According to a meta-analysis of 102 prospective studies involving 698,782 individuals, the adjusted hazard ratio was 2.27 (95% CI, 1.95–2.65) for ischemic stroke among those with type 2 diabetes compared to those without.^[81]^ Additionally, a study has demonstrated that female patients with type 2 diabetes are significantly more vulnerable to stroke than their male counterparts.^[90]^ Age, smoking, obesity, hypertension, high-density lipoprotein cholesterol, HbA1c, history of vascular disease, heart failure, and atrial fibrillation are known to be associated with CeVD based on multiple studies.^[91,92]^ A. Rocco et al conducted a review of microalbuminuria in relation to CeVD and found that it may be predictive of stroke, although further studies are necessary.^[93]^ Recent evidence has shown that the microbiota plays a critical role in the onset of stroke.^[88]^ Moreover, an increasing body of research has examined the influence of genetic variations and epigenetic alterations in the onset of CeVD.^[94,95]^ For example, PITX2 and ZFHX3, which are associated with atrial fibrillation, have been identified as contributors to the susceptibility to ischemic stroke among Caucasians.^[96,97]^ The advent of genome-wide association studies enabled the identification of more novel genetic variants associated with the risk of CeVD.

The mechanisms by which the risk factors mentioned above influence the occurrence of CeVD are different and not fully understood. However, the common point is that these factors are able to reduce the onset time of overt atherosclerosis through promote endothelial dysfunction.^[98,99]^ Atherosclerosis is the main cause of CeVD development, and its related pathogenesis of CeVD is complex and multifactorial. Of these factors, oxidative stress and inflammatory pathways have been extensively investigated as key players.^[100–102]^

### 3.3. Peripheral artery disease

In addition to CAD and CeVD, another common macrovascular complication in patients with diabetes is PAD. PAD, also known as peripheral vascular disease (PVD), is characterized by atherosclerotic occlusion in the lower extremities. The most common symptom of PAD is claudication, which is characterized by cramping, pain, or pain in the lower leg, thigh, or buttock with exertion, which relives with rest.^[103]^ The ankle-brachial index (ABI) is used to diagnose and grade the severity of PAD, with a cutoff value of 0.9 indicating arterial occlusion. However, ABI has limited sensitivity in patients with diabetes compared to non-diabetic patients. High ABI levels may indicate arterial occlusion in patients with diabetes, leading to the underdiagnosis of PAD in this population.^[104,105]^

PVD has emerged as a global health concern, affecting over 200 million individuals worldwide. In the United States, estimates suggest that more than 8 million patients aged 40 years and above have low ABI levels (<0.9), with a quarter of these patients experiencing severe PAD (ABI <0.7).^[106]^ PAD is significantly associated with increased mortality rates, higher risk of lower extremity amputations, and elevated rates of cardiovascular events.^[103,107]^

DM is associated with a higher risk of PAD, with a 2 to 4-fold increase compared to the risk of CAD or stroke.^[16,108,109]^ Several risk factors have been identified for the development of PVD in individuals with diabetes, including smoking, obesity, hypertension, hypercholesterolemia, and dyslipidemia.^[110,111]^ A cross-sectional study of 271 patients with diabetes found a significant association between PVD and smoking, hypertension, hypercholesterolemia, and obesity.^[112]^ Other factors that have been linked to an increased risk of PVD in individuals with diabetes include the duration of diabetes, degree of hyperglycemia, increasing age, male gender, elevated serum lipoprotein levels, insulin resistance, elevated serum fibrinogen levels, microalbuminuria, and increased levels of intercellular adhesion molecules.^[111]^

The mechanism of PAD development and progression in diabetes is similar to that of other macrovascular complications, including derangements in the vessel wall through the promotion of vascular inflammation, endothelial cell dysfunction, abnormalities in blood cells, and factors affecting hemostasis.^[113]^ Of note, there is a significant interplay between different mechanisms: for example, impaired NO production can affect inflammation, endothelial function, and arteriogenesis, whereas increased ROS can lead to platelet and endothelial dysfunction.^[107]^

## 4. Management of diabetes complications

The prevalence of diabetes is on the rise, and its associated complications are widespread. Therefore, it is crucial to provide specific recommendations for diabetes screening and management, as early intervention can effectively delay the disease progression and improve outcomes. For example, timely detection and treatment can prevent as much as 98% of diabetes-related visual impairments.^[114]^

Early detection of diabetes is imperative in preventing and managing its severe and irreversible complications, as many patients with diabetes remain unaware of their ailment. This is because diabetes often begins with few or no significant signs or symptoms. Consequently, these undiagnosed patients suffer from delayed diagnoses (4–7 years) and the disease serious consequences and complications.^[115,116]^ Presently, several methods and strategies are employed to conduct diabetes screening programs focused on high-risk groups (e.g., older adults or those with defined risk factors such as overweight or positive family history) to enhance screening program efficacy and affordability. And the tests of population-based screening for diabetes included capillary fasting blood glucose, venous plasma glucose, and HbA1c.^[117,118]^ However, these tests have their own limitations, so it is necessary to choose according to the specific situation. In addition, due to the high heterogeneity of diabetes,^[119]^ accurate classification of patients with diabetes and identification of high-risk groups with different complications are also of great significance for the management of diabetic patients. For example, 1 study identified 3 distinct subgroups of type 2 diabetes from topology-based patient-patient networks. Subtype 1 was characterized by DN and DR; subtype 2 was enriched for cancer malignancy and CAD; and subtype 3 was associated most strongly with CAD, and neurological diseases.^[120]^ Similarly, Emma Ahlqvist et al performed a data-driven cluster analysis in patients with newly diagnosed diabetes (n = 8980) based on 6 variables (glutamic acid decarboxylase antibodies, age at diagnosis, body mass index, HbA1c, and homoeostatic model assessment 2 estimates of β-cell function and insulin resistance) and correlated with prospective data from patient records on occurrence of complications and prescription of medication. Subsequently, the study identified 5 reproducible populations of diabetic patients with significantly different patient characteristics and risks of diabetic complications. Patients in cluster 3 (who are most resistant to insulin) have a significantly higher risk of DN than those in groups 4 and 5, despite they receive similar diabetes treatment.^[121]^ Precise stratification to identify individuals with increased risk of complications at diagnosis could provide a powerful tool for individualized treatment planning.

### 4.1. Lifestyle modification

Risk factors, such as hypertension, obesity, and hyperglycemia, are closely associated with the development and progression of diabetic vascular complications. Lifestyle modification and professional medical prevention targeting these risk factors will yield positive outcomes. Lifestyle modification involves reducing the intake of sugar-sweetened beverages, engaging in moderate exercise, and quitting smoking. A meta-analysis study revealed that increased consumption of sugar- and artificially-sweetened beverages is linked to the risk of type 2 diabetes, CVDs, and all-cause mortality.^[122]^ Therefore, reducing the intake of sugar-sweetened beverages may be a viable option to lower the risk of diabetic complications. Another study demonstrated that patients who do not exercise have relatively poor control of vascular complications in type 2 diabetes and are more likely to develop vascular complications. Therefore, exercise may help patients with diabetes prevent vascular complications.^[123]^ Furthermore, growing evidence highlights the impact of smoking on diabetic complications,^[124,125]^ and suggests that smoking cessation is crucial not only to prevent macrovascular complications in diabetes but also to limit microvascular disease.^[126,127]^

### 4.2. Blood glucose, lipids, and pressure control

Epidemiological studies have consistently shown that lower levels of blood glucose, blood pressure, and cholesterol are associated with reduced risk of microvascular and macrovascular complications in individuals with diabetes.^[21,128,129]^ In an attempt to confirm these relationships, clinical trials have targeted each individual risk factor or used multiple interventions to lower all 3 parameters simultaneously.^[130–133]^ For instance, the UK prospective diabetes study demonstrated that treating hypertension reduces macrovascular disease in individuals with type 2 diabetes.^[134]^ Moreover, lipid-lowering agents, especially statins, have been shown to reduce macrovascular disease in individuals with diabetes in numerous studies. The Steno-2 clinical trial unequivocally demonstrated the benefits of aggressive control of all 3 parameters on vascular outcomes in individuals with diabetes. In this study, 160 patients with type 2 diabetes and microalbuminuria were enrolled and randomly assigned to either conventional treatment or intensive treatment, which included behavior modification and pharmacologic therapy targeting hyperglycemia, hypertension, dyslipidemia, and microalbuminuria, along with secondary prevention of CVD with aspirin. After a mean follow-up of 7.8 years, the intensive-treatment group had significantly greater reductions in glycated hemoglobin values, blood pressure, serum cholesterol and triglyceride levels, and urinary albumin excretion rate than the conventional-treatment group, resulting in a 50% reduction in the risk of cardiovascular and microvascular events.^[130]^ However, intensive therapy targeting only blood glucose is associated with a significantly increased risk of hypoglycemia, including those requiring pharmacologic intervention for recovery. The ADVANCE, ACCORD, and VADT trials all showed no significant difference in the incidence of macrovascular complications, cardiovascular mortality, and overall mortality between intensive glucose control and standard treatment control groups, but a higher incidence of severe hypoglycemia in the intensive glucose control group.^[132,135,136]^ Similarly, lowering systolic blood pressure below 120 mm Hg in individuals at high cardiovascular risk with diabetes is associated with significant excess adverse events, which would limit the utility of such an intervention.^[137]^

### 4.3. Screening and treatment of specific microvascular complications

#### 4.3.1. *Diabetic retinopathy*.

Vascular complications in patients with diabetes require prompt diagnosis and timely intervention to prevent or delay the onset of end-stage diseases such as blindness, renal replacement therapy, or amputation. To screen for DR, visual acuity should be assessed and retinopathy evaluated.^[138]^ Various screening methods are available, including direct ophthalmoscopy, dilated slit lamp bio-microscopy with a hand-held lens, tele-retinal screening, mydriatic or non-mydriatic retinal photography, and retinal video recording. Retinal still photography, both mydriatic and non-mydriatic, has been identified as a cost-effective DR screening strategy.^[138,139]^ The International Council of Ophthalmology Guidelines recommend that examiners evaluate patients’ best-corrected visual acuity and obtain a comprehensive history of diabetes, including HbA1c, blood pressure profile, lipid profile, smoking status, and other diabetes-related complications, to make a more accurate assessment of the patient condition.^[23]^ Currently, anti-vascular endothelial growth factors (e.g., aflibercept, ranibizumab, or bevacizumab) are the primary treatment to improve vision in diabetic macular edema.^[140]^ Therefore, researchers have tried to explore alternative novel therapeutics for DR. For instance, Yue Cai et al proposed that modulating the gut microbiota may prevent and treat DR as novel strategies based on the relationship between gut microbiota and DR.^[26]^

#### 4.3.2. Diabetic nephropathy.

Microalbuminuria is often the first sign of DN.^[141]^ Therefore, screening and diagnosis of DN rely on assessing albuminuria in a spot urine sample. Patients with type 2 diabetes have a higher prevalence of microalbuminuria compared to those with type 1 diabetes. As a result, the American Diabetes Association recommends initiating screening for DN at the time of diagnosis for type 2 diabetes patients since about 7% of them already have microalbuminuria.^[142]^ For type 1 diabetes patients, the first screening is recommended 5 years after diagnosis.^[142]^ However, microalbuminuria is not uncommon in type 1 diabetes patients before 5 years, with a prevalence of 18%, particularly in those with poor glycemic and lipid control and high normal blood pressure levels.^[143]^ Therefore, screening for microalbuminuria in type 1 diabetes patients is recommended 1 year after diagnosis.^[37]^ The screening must be repeated annually for both type 1 and type 2 diabetic patients.^[142]^ Accurate measurement of kidney function is critical in screening for kidney disease, which is mainly achieved by assessing the GFR. Some patients with diabetes may have decreased GFR even in the presence of normal urinary albumin excretion.^[144,145]^ While direct measurement of GFR through clearance of exogenous filtration markers such as iohexol or inulin is accurate, its application outside clinical research is limited due to time-consuming and cumbersome limitations.^[146]^ In contrast, GFR is mainly measured indirectly by assessing the clearance of endogenous filtration markers such as serum creatinine or cystatin C.^[147]^ In addition to albuminuria and GFR, the kidney biopsy may be performed for specific indications, such as significant and persistent proteinuria and a family history of non-diabetic forms of kidney disease.^[36]^ However, the biopsy should be performed with careful consideration of the risks and benefits of the procedure.

The management of DN primarily aims to prevent renal injury, reduce the risk of end-stage kidney disease, and lower the chances of CVD and mortality. Non-pharmacological interventions such as weight loss, increased physical activity, reduced dietary sodium intake, and smoking cessation are crucial components of any strategy to improve outcomes in patients with DN.^[148,149]^ While the standard therapy for DN focuses on controlling glucose and blood pressure, with the goal of reducing albuminuria and halting DN progression, this approach can only slow down the rate of development and cannot stop or reverse the disease.^[150]^ Therefore, novel therapeutic strategies are necessary. Currently, new drugs targeting oxidative stress and inflammation, such as mineralocorticoid receptor antagonists and endothelin receptor antagonists, have garnered significant attention.^[36]^

#### 4.3.3. Diabetic neuropathy.

Amputation due to diabetic neuropathy has a devastating effect on the quality of life and is associated with an alarmingly low life expectancy. Therefore, it is particularly important to reduce the incidence and delay the progression of diabetic neuropathy through screening and early intervention. Screening for diabetic neuropathy is recommended for all patients with type 2 diabetes at diagnosis and for those with type 1 diabetes starting 5 years after diagnosis. After the initial screening, screening was performed annually thereafter.^[151]^ Common screening methods for peripheral neuropathy include tests of peripheral sensation by pinprick of the foot, ankle reflexes, vibration sensation via tuning fork, and examination of proprioception, or awareness of body positioning. And, Screening for cardiac autonomic neuropathy is more complex than tests of peripheral neuropathy and may include measures of heart-rate variability and quantification of the QT interval via electrocardiography.^[152]^ About 50% of diabetic patients are affected by diabetic peripheral neuropathy. To date, treatments have not been shown to successfully repair the nerve damage that underlies diabetic neuropathy. For instance, several approved agents for the relief of pain caused by diabetic neuropathy, but do not affect the pathologic process.^[153]^ Similarly, good glycemic control can slow the progression of diabetic peripheral neuropathy, and only in type 1 diabetic patients.^[154]^ Therefore, there is an urgent need to develop novel drugs that can effectively treat nerve injury.

## 5. Conclusions

This manuscript provides a brief review of the epidemiology, risk factors, pathogenesis, and management of microvascular and macrovascular complications associated with diabetes. Microvascular complications are a significant cause of increased morbidity and reduced quality of life in patients with diabetes. While macrovascular diseases significantly increase the mortality rate of patients with diabetes, especially those with type 2 diabetes. Common risk factors for diabetes and its associated macrovascular and microvascular complications include hyperglycemia, hypertension, hyperlipidemia, duration of diabetes, overweight, and smoking. Therefore, managing diabetic vascular complications requires intensive control of glucose, blood pressure, and lipid levels, early screening and diagnosis of diabetes, appropriate exercise, and smoking cessation. Furthermore, accurate risk stratification of patients with diabetic vascular complications and specific screening for different complications are essential for early intervention to delay progression and improve outcomes. Finally, the genetics of vascular complications associated with diabetes have gained broad attention, and recent advances in genome-wide technologies will help contribute to improving the risk prediction and prevention of complications.

## Author contributions

**Conceptualization:** Yongxia Lu, Wei Wang, Sufang Li.

**Investigation:** Yongxia Lu, Wei Wang, Jingyu Liu.

**Supervision:** Sufang Li.

**Writing – original draft:** Yongxia Lu, Wei Wang, Min Xie.

**Writing – review & editing:** Yongxia Lu, Wei Wang, Jingyu Liu, Min Xie, Qiang Liu, Sufang Li.

## References

[1] Saeedi P Petersohn I Salpea P. Global and regional diabetes prevalence estimates for 2019 and projections for 2030 and 2045: results from the International Diabetes Federation Diabetes Atlas. Diabetes Res Clin Pract. 2019;157:107843. 31518657 10.1016/j.diabres.2019.107843

[2] Ling W Huang Y Huang YM. Global trend of diabetes mortality attributed to vascular complications, 2000–2016. Cardiovasc Diabetol. 2020;19:182. 33081808 10.1186/s12933-020-01159-5 PMC7573870

[3] Khalil H. Diabetes microvascular complications-a clinical update. Diabetes Metab Syndr. 2017;11(Suppl 1):S133–9. 27993541 10.1016/j.dsx.2016.12.022

[4] Hu M Ma Q Liu B. Long non-coding RNAs in the pathogenesis of diabetic kidney disease. Front Cell Dev Biol. 2022;10:845371. 35517509 10.3389/fcell.2022.845371 PMC9065414

[5] Chen Y He Y Zhou H. The potential role of lncRNAs in diabetes and diabetic microvascular complications. Endocr J. 2020;67:659–68. 32404556 10.1507/endocrj.EJ19-0574

[6] Stefan N Cusi K. A global view of the interplay between non-alcoholic fatty liver disease and diabetes. Lancet Diabetes Endocrinol. 2022;10:284–96. 35183303 10.1016/S2213-8587(22)00003-1

[7] Lv WS Sun RX Gao YY. Nonalcoholic fatty liver disease and microvascular complications in type 2 diabetes. World J Gastroenterol. 2013;19:3134–42. 23716995 10.3748/wjg.v19.i20.3134 PMC3662955

[8] Wen X Zhou X Chen D. Association between non-alcoholic fatty liver disease and diabetes-related microvascular complications: a retrospective cross-sectional study of hospitalized patients. Endocr Pract. 2022;28:304–9. 33601024 10.1016/j.eprac.2021.02.004

[9] Targher G Lonardo A Byrne CD. Nonalcoholic fatty liver disease and chronic vascular complications of diabetes mellitus. Nat Rev Endocrinol. 2018;14:99–114. 29286050 10.1038/nrendo.2017.173

[10] Stitt AW Curtis TM Chen M. The progress in understanding and treatment of diabetic retinopathy. Prog Retin Eye Res. 2016;51:156–86. 26297071 10.1016/j.preteyeres.2015.08.001

[11] Yau JW Rogers SL Kawasaki R. Global prevalence and major risk factors of diabetic retinopathy. Diabetes Care. 2012;35:556–64. 22301125 10.2337/dc11-1909 PMC3322721

[12] Zhang X Saaddine JB Chou CF. Prevalence of diabetic retinopathy in the United States, 2005-2008. JAMA. 2010;304:649–56. 20699456 10.1001/jama.2010.1111 PMC2945293

[13] Cheung N Mitchell P Wong TY. Diabetic retinopathy. Lancet (London, England). 2010;376:124–36. 20580421 10.1016/S0140-6736(09)62124-3

[14] Fong DS Aiello LP Ferris F III. Reviews/Commentaries/ADA Statements-Technical Review-Diabetic retinopathy. Diabetes Care. 2004;27:2540–53. 15451934 10.2337/diacare.27.10.2540

[15] Flaxman SR Bourne RRA Resnikoff S. Global causes of blindness and distance vision impairment 1990-2020: a systematic review and meta-analysis. Lancet Glob Health. 2017;5:e1221–34. 29032195 10.1016/S2214-109X(17)30393-5

[16] Beckman JA Creager MA. Vascular complications of diabetes. Circ Res. 2016;118:1771–85. 27230641 10.1161/CIRCRESAHA.115.306884

[17] Sun Q Jing Y Zhang B. The risk factors for diabetic retinopathy in a Chinese population: a cross-sectional study. J Diabetes Res. 2021;2021:5340453. 33575359 10.1155/2021/5340453 PMC7861953

[18] Hainsworth DP Bebu I Aiello LP. Risk factors for retinopathy in type 1 diabetes: the DCCT/EDIC study. Diabetes Care. 2019;42:875–82. 30833368 10.2337/dc18-2308 PMC6489114

[19] Song KH Jeong JS Kim MK. Discordance in risk factors for the progression of diabetic retinopathy and diabetic nephropathy in patients with type 2 diabetes mellitus. J Diabetes Investig. 2019;10:745–52. 10.1111/jdi.12953 PMC6497586 30300472

[20] Tarasewicz D Conell C Gilliam LK. Quantification of risk factors for diabetic retinopathy progression. Acta Diabetol. 2023;60:363–9. 36527502 10.1007/s00592-022-02007-6

[21] Stratton IM Adler AI Neil HA. Association of glycaemia with macrovascular and microvascular complications of type 2 diabetes (UKPDS 35): prospective observational study. BMJ. 2000;321:405–12. 10938048 10.1136/bmj.321.7258.405 PMC27454

[22] Stratton IM Kohner EM Aldington SJ. UKPDS 50: risk factors for incidence and progression of retinopathy in Type II diabetes over 6 years from diagnosis. Diabetologia. 2001;44:156–63. 11270671 10.1007/s001250051594

[23] Ting DS Cheung GC Wong TY. Diabetic retinopathy: global prevalence, major risk factors, screening practices and public health challenges: a review. Clin Exp Ophthalmol. 2016;44:260–77. 26716602 10.1111/ceo.12696

[24] Vuori N Sandholm N Kumar A. CACNB2 is a novel susceptibility gene for diabetic retinopathy in type 1 diabetes. Diabetes. 2019;68:2165–74. 31439644 10.2337/db19-0130 PMC6804633

[25] Estacio RO McFarling E Biggerstaff S. Overt albuminuria predicts diabetic retinopathy in Hispanics with NIDDM. Am J Kid Dis. 1998;31:947–53. 9631838 10.1053/ajkd.1998.v31.pm9631838

[26] Cai Y Kang Y. Gut microbiota and metabolites in diabetic retinopathy: insights into pathogenesis for novel therapeutic strategies. Biomed Pharmacother. 2023;164:114994. 37301133 10.1016/j.biopha.2023.114994

[27] Tarr JM Kaul K Chopra M. Pathophysiology of diabetic retinopathy. ISRN Ophthalmol. 2013;2013:343560. 24563789 10.1155/2013/343560 PMC3914226

[28] Giacco F Brownlee M. Oxidative stress and diabetic complications. Circ Res. 2010;107:1058–70. 21030723 10.1161/CIRCRESAHA.110.223545 PMC2996922

[29] Lin WJ Kuang HY. Oxidative stress induces autophagy in response to multiple noxious stimuli in retinal ganglion cells. Autophagy. 2014;10:1692–701. 25207555 10.4161/auto.36076 PMC4198355

[30] Rodríguez ML Pérez S Mena-Mollá S. Oxidative stress and microvascular alterations in diabetic retinopathy: future therapies. Oxid Med Cell Longevity. 2019;2019:4940825. 10.1155/2019/4940825 PMC6878793 31814880

[31] Das T Jayasudha R Chakravarthy S. Alterations in the gut bacterial microbiome in people with type 2 diabetes mellitus and diabetic retinopathy. Sci Rep. 2021;11:2738. 33531650 10.1038/s41598-021-82538-0 PMC7854632

[32] Singh V Yeoh BS Vijay-Kumar M. Gut microbiome as a novel cardiovascular therapeutic target. Curr Opin Pharmacol. 2016;27:8–12. 26828626 10.1016/j.coph.2016.01.002 PMC4808470

[33] Jiao J Yu H Yao L. Recent insights into the role of gut microbiota in diabetic retinopathy. J Inflammation Res. 2021;14:6929–38. 10.2147/JIR.S336148 PMC8687677 34938095

[34] Huang Y Wang Z Ma H. Dysbiosis and implication of the gut microbiota in diabetic retinopathy. Front Cell Infect Microbiol. 2021;11:646348. 33816351 10.3389/fcimb.2021.646348 PMC8017229

[35] Thakur PS Aggarwal D Takkar B. Evidence suggesting the role of gut dysbiosis in diabetic retinopathy. Invest Ophthalmol Vis Sci. 2022;63:21. 10.1167/iovs.63.8.21 PMC9339698 35877085

[36] Samsu N. Diabetic Nephropathy: challenges in pathogenesis, diagnosis, and treatment. Biomed Res Int. 2021;2021:1497449. 34307650 10.1155/2021/1497449 PMC8285185

[37] Gross JL de Azevedo MJ Silveiro SP. Diabetic nephropathy: diagnosis, prevention, and treatment. Diabetes Care. 2005;28:164–76. 15616252 10.2337/diacare.28.1.164

[38] Zhang XX Kong J Yun K. Prevalence of diabetic nephropathy among patients with type 2 diabetes mellitus in China: a meta-analysis of observational studies. J Diabetes Res. 2020;2020:2315607. 32090116 10.1155/2020/2315607 PMC7023800

[39] Al-Rubeaan K Youssef AM Subhani SN. Diabetic nephropathy and its risk factors in a society with a type 2 diabetes epidemic: a Saudi National Diabetes Registry-based study. PLoS One. 2014;9:e88956. 24586457 10.1371/journal.pone.0088956 PMC3931705

[40] Li S Xie H Shi Y. Prevalence of diabetic nephropathy in the diabetes mellitus population: a protocol for systematic review and meta-analysis. Medicine (Baltimore). 2022;101:e31232. 36281143 10.1097/MD.0000000000031232 PMC9592388

[41] Gregg EW Li Y Wang J. Changes in diabetes-related complications in the United States, 1990–2010. N Engl J Med. 2014;370:1514–23. 24738668 10.1056/NEJMoa1310799

[42] Wu H Eggleston KN Zhong J. How do type 2 diabetes mellitus (T2DM)-related complications and socioeconomic factors impact direct medical costs? A cross-sectional study in rural Southeast China. BMJ Open. 2018;8:e020647. 10.1136/bmjopen-2017-020647 PMC6224711 30389755

[43] Agarwal N Sengar NS Jain PK. Nephropathy in newly diagnosed type 2 diabetics with special stress on the role of hypertension. J Assoc Physicians India. 2011;59:145–7. 21751621

[44] Shestakova MV Koshel LV Vagodin VA. [Risk factors of diabetic nephropathy progression in patients with a long history of diabetic mellitus as shown by a retrospective analysis]. Ter Arkh. 2006;78:60–64. 16889052

[45] Lim A. Diabetic nephropathy - complications and treatment. Int J Nephrol Renovasc Dis. 2014;7:361–81. 25342915 10.2147/IJNRD.S40172 PMC4206379

[46] Scott LJ Warram JH Hanna LS. A nonlinear effect of hyperglycemia and current cigarette smoking are major determinants of the onset of microalbuminuria in type 1 diabetes. Diabetes. 2001;50:2842–9. 11723069 10.2337/diabetes.50.12.2842

[47] Mooyaart AL Valk EJ van Es LA. Genetic associations in diabetic nephropathy: a meta-analysis. Diabetologia. 2011;54:544–53. 21127830 10.1007/s00125-010-1996-1 PMC3034040

[48] Trevisan R Vedovato M Mazzon C. Concomitance of diabetic retinopathy and proteinuria accelerates the rate of decline of kidney function in type 2 diabetic patients. Diabetes Care. 2002;25:2026–31. 12401751 10.2337/diacare.25.11.2026

[49] Xiong Y Zhou L. The signaling of cellular senescence in diabetic nephropathy. Oxid Med Cell Longevity. 2019;2019:7495629. 10.1155/2019/7495629 PMC6794967 31687085

[50] Han Q Zhu H Chen X. Non-genetic mechanisms of diabetic nephropathy. Front Med. 2017;11:319–32. 28871454 10.1007/s11684-017-0569-9

[51] Kopel J Pena-Hernandez C Nugent K. Evolving spectrum of diabetic nephropathy. World J Diabetes. 2019;10:269–79. 31139314 10.4239/wjd.v10.i5.269 PMC6522757

[52] Grover M Makkar R Sehgal A. Etiological aspects for the occurrence of diabetic neuropathy and the suggested measures. Neurophysiology. 2020;52:159–68.

[53] Callaghan BC Kerber KA Lisabeth LL. Role of neurologists and diagnostic tests on the management of distal symmetric polyneuropathy. JAMA Neurol. 2014;71:1143–9. 25048157 10.1001/jamaneurol.2014.1279 PMC4266395

[54] Beghi E Monticelli L. Chronic symmetric symptomatic polyneuropathy in the elderly: a field screening investigation of risk factors for polyneuropathy in two Italian communities. J Clin Epidemiol. 1998;51:697–702. 9743318 10.1016/s0895-4356(98)00039-0

[55] Visser NA Notermans NC Linssen RS. Incidence of polyneuropathy in Utrecht, the Netherlands. Neurology. 2015;84:259–64. 25503982 10.1212/WNL.0000000000001160

[56] Partanen J Niskanen L Lehtinen J. Natural history of peripheral neuropathy in patients with non-insulin-dependent diabetes mellitus. N Engl J Med. 1995;333:89–94. 7777034 10.1056/NEJM199507133330203

[57] Tesfaye S Chaturvedi N Eaton SE. Vascular risk factors and diabetic neuropathy. N Engl J Med. 2005;352:341–50. 15673800 10.1056/NEJMoa032782

[58] Andersen ST Witte DR Dalsgaard E-M. Risk factors for incident diabetic polyneuropathy in a cohort with screen-detected type 2 diabetes followed for 13 years: ADDITION-Denmark. Diabetes Care. 2018;41:1068–75. 29487078 10.2337/dc17-2062

[59] Sloan G Shillo P Selvarajah D. A new look at painful diabetic neuropathy. Diabetes Res Clin Pract. 2018;144:177–91. 30201394 10.1016/j.diabres.2018.08.020

[60] Pathak R Sachan N Chandra P. Mechanistic approach towards diabetic neuropathy screening techniques and future challenges: a review. Biomed Pharmacother. 2022;150:113025. 35658222 10.1016/j.biopha.2022.113025

[61] Drel VR Lupachyk S Shevalye H. New therapeutic and biomarker discovery for peripheral diabetic neuropathy: PARP inhibitor, nitrotyrosine, and tumor necrosis factor-{alpha}. Endocrinology. 2010;151:2547–55. 20357221 10.1210/en.2009-1342 PMC2875829

[62] Oates PJ. Polyol pathway and diabetic peripheral neuropathy. Int Rev Neurobiol. 2002;50:325–92. 12198816 10.1016/s0074-7742(02)50082-9

[63] Dal Canto E Ceriello A Rydén L. Diabetes as a cardiovascular risk factor: an overview of global trends of macro and micro vascular complications. Eur J Prev cardiol. 2019;26(2_suppl):25–32. 31722562 10.1177/2047487319878371

[64] Tang H Fang Z Wang T. Meta-analysis of effects of sodium-glucose cotransporter 2 inhibitors on cardiovascular outcomes and all-cause mortality among patients with type 2 diabetes mellitus. Am J Cardiol. 2016;118:1774–80. 27666177 10.1016/j.amjcard.2016.08.061

[65] Romon I Fosse S Eschwège E. Prevalence of macrovascular complications and cardiovascular risk factors in people treated for diabetes and living in France: the ENTRED study 2001. Diabetes Metab. 2008;34:140–7. 18304854 10.1016/j.diabet.2007.11.002

[66] Lee CH Wu YL Kuo JF. Prevalence of diabetic macrovascular complications and related factors from 2005 to 2014 in Taiwan: a nationwide survey. J FormosMed Assoc. 2019;118(Suppl 2):S96–S102. 10.1016/j.jfma.2019.08.035 31540817

[67] Fhärm E Cederholm J Eliasson B. Time trends in absolute and modifiable coronary heart disease risk in patients with Type 2 diabetes in the Swedish National Diabetes Register (NDR) 2003-2008. Diabet Med. 2012;29:198–206. 21883434 10.1111/j.1464-5491.2011.03425.x

[68] Gregg EW Cheng YJ Saydah S. Trends in death rates among U.S. adults with and without diabetes between 1997 and 2006: findings from the National Health Interview Survey. Diabetes Care. 2012;35:1252–7. 22619288 10.2337/dc11-1162 PMC3357247

[69] Ford ES. Trends in the risk for coronary heart disease among adults with diagnosed diabetes in the U.S.: findings from the National Health and Nutrition Examination Survey, 1999-2008. Diabetes Care. 2011;34:1337–43. 21505207 10.2337/dc10-2251 PMC3114334

[70] Grant PJ. Diabetes mellitus as a prothrombotic condition. J Intern Med. 2007;262:157–72. 17645584 10.1111/j.1365-2796.2007.01824.x

[71] Huang D Refaat M Mohammedi K. Macrovascular complications in patients with diabetes and prediabetes. Biomed Res Int. 2017;2017:7839101. 29238721 10.1155/2017/7839101 PMC5697393

[72] Lombardi R Airaghi L Targher G. Liver fibrosis by FibroScan(®) independently of established cardiovascular risk parameters associates with macrovascular and microvascular complications in patients with type 2 diabetes. Liver Int. 2020;40:347–54. 31612634 10.1111/liv.14274

[73] Mantovani A Dalbeni A Beatrice G. Non-Alcoholic fatty liver disease and risk of macro- and microvascular complications in patients with type 2 diabetes. J Clin Med. 2022;11:968. 35207239 10.3390/jcm11040968 PMC8878156

[74] Viswanathan V Kadiri M Medimpudi S. Association of non-alcoholic fatty liver disease with diabetic microvascular and macrovascular complications in South Indian diabetic subjects. Int J Diabetes Dev Countries. 2010;30:208.

[75] Stefan N Schick F Birkenfeld AL. The role of hepatokines in NAFLD. Cell Metab. 2023;35:236–52. 36754018 10.1016/j.cmet.2023.01.006 PMC10157895

[76] Beckman JA Paneni F Cosentino F. Diabetes and vascular disease: pathophysiology, clinical consequences, and medical therapy: part II. Eur Heart J. 2013;34:2444–52. 23625211 10.1093/eurheartj/eht142

[77] Cade WT. Diabetes-related microvascular and macrovascular diseases in the physical therapy setting. Phys Ther. 2008;88:1322–35. 18801863 10.2522/ptj.20080008 PMC2579903

[78] Buyken AE von Eckardstein A Schulte H. Type 2 diabetes mellitus and risk of coronary heart disease: results of the 10-year follow-up of the PROCAM study. Eur J Cardiovasc Prev Rehab. 2007;14:230–6. 10.1097/HJR.0b013e3280142037 17446801

[79] Malakar AK Choudhury D Halder B. A review on coronary artery disease, its risk factors, and therapeutics. J Cell Physiol. 2019;234:16812–23. 30790284 10.1002/jcp.28350

[80] Einarson TR Acs A Ludwig C. Prevalence of cardiovascular disease in type 2 diabetes: a systematic literature review of scientific evidence from across the world in 2007–2017. Cardiovasc Diabetol. 2018;17:83. 29884191 10.1186/s12933-018-0728-6 PMC5994068

[81] Sarwar N Gao P Seshasai SR. Diabetes mellitus, fasting blood glucose concentration, and risk of vascular disease: a collaborative meta-analysis of 102 prospective studies. Lancet (London, England). 2010;375:2215–22. 20609967 10.1016/S0140-6736(10)60484-9 PMC2904878

[82] Haffner SM Lehto S Rönnemaa T. Mortality from coronary heart disease in subjects with type 2 diabetes and in nondiabetic subjects with and without prior myocardial infarction. N Engl J Med. 1998;339:229–34. 9673301 10.1056/NEJM199807233390404

[83] Bulugahapitiya U Siyambalapitiya S Sithole J. Is diabetes a coronary risk equivalent? Systematic review and meta-analysis. Diabet Med. 2009;26:142–8. 19236616 10.1111/j.1464-5491.2008.02640.x

[84] Anand SS Islam S Rosengren A. Risk factors for myocardial infarction in women and men: insights from the INTERHEART study. Eur Heart J. 2008;29:932–40. 18334475 10.1093/eurheartj/ehn018

[85] Cavender MA Scirica BM Bonaca MP. Vorapaxar in patients with diabetes mellitus and previous myocardial infarction: findings from the thrombin receptor antagonist in secondary prevention of atherothrombotic ischemic events-TIMI 50 trial. Circulation. 2015;131:1047–53. 25681464 10.1161/CIRCULATIONAHA.114.013774 PMC4365950

[86] Tonyan ZN Nasykhova YA Danilova MM. Genetics of macrovascular complications in type 2 diabetes. World J Diabetes. 2021;12:1200–19. 34512887 10.4239/wjd.v12.i8.1200 PMC8394234

[87] Shi M Tang R Huang F. Cardiovascular disease in patients with type 1 diabetes: early evaluation, risk factors and possible relation with cardiac autoimmunity. Diabetes Metab Res Rev. 2021;37:e3423. 33252830 10.1002/dmrr.3423

[88] Tonomura S Ihara M Friedland RP. Microbiota in cerebrovascular disease: a key player and future therapeutic target. J Cereb Blood Flow Metab. 2020;40:1368–80. 32312168 10.1177/0271678X20918031 PMC7308516

[89] Virani SS Alonso A Aparicio HJ. Heart disease and stroke statistics-2021 update: a report from the American Heart Association. Circulation. 2021;143:e254–743. 33501848 10.1161/CIR.0000000000000950 PMC13036842

[90] Guo L Yu M Zhong J. Stroke risk among patients with type 2 diabetes mellitus in Zhejiang: a population-based prospective study in China. Int J Endocrinol. 2016;2016:1–8. 10.1155/2016/6380620 PMC4923572 27403161

[91] Proietti M Mairesse GH Goethals P. Cerebrovascular disease, associated risk factors and antithrombotic therapy in a population screening cohort: insights from the Belgian Heart Rhythm Week programme. Eur J prev Cardiol. 2017;24:328–34. 27909151 10.1177/2047487316682349

[92] Oe M Fujihara K Harada-Yamada M. Impact of prior cerebrovascular disease and glucose status on incident cerebrovascular disease in Japanese. Cardiovasc Diabetol. 2021;20:174. 34479567 10.1186/s12933-021-01367-7 PMC8417951

[93] Rocco A Heerlein K Diedler J. Microalbuminuria in cerebrovascular disease: a modifiable risk factor? Int J Stroke. 2010;5:30–4. 20088991 10.1111/j.1747-4949.2009.00398.x

[94] Griessenauer CJ Farrell S Sarkar A. Genetic susceptibility to cerebrovascular disease: a systematic review. J Cereb Blood Flow Metab. 2018;38:1853–71. 30182779 10.1177/0271678X18797958 PMC6259318

[95] Della-Morte D Pacifici F Rundek T. Genetic susceptibility to cerebrovascular disease. Curr Opin Lipidol. 2016;27:187–95. 26959706 10.1097/MOL.0000000000000275 PMC5138857

[96] Gretarsdottir S Thorleifsson G Manolescu A. Risk variants for atrial fibrillation on chromosome 4q25 associate with ischemic stroke. Ann Neurol. 2008;64:402–9. 18991354 10.1002/ana.21480

[97] Gudbjartsson DF Holm H Gretarsdottir S. A sequence variant in ZFHX3 on 16q22 associates with atrial fibrillation and ischemic stroke. Nat Genet. 2009;41:876–8. 19597491 10.1038/ng.417 PMC2740741

[98] Scicchitano P Cortese F Gesualdo M. The role of endothelial dysfunction and oxidative stress in cerebrovascular diseases. Free Radic Res. 2019;53:579–95. 31106620 10.1080/10715762.2019.1620939

[99] Ciccone MM Miniello V Marchioli R. Morphological and functional vascular changes induced by childhood obesity. Eur J Cardiovasc Prev Rehab. 2011;18:831–5. 10.1177/1741826711398180 21450599

[100] Ross R. Atherosclerosis--an inflammatory disease. N Engl J Med. 1999;340:115–26. 9887164 10.1056/NEJM199901143400207

[101] Orellana-Urzúa S Rojas I Líbano L. Pathophysiology of ischemic stroke: role of oxidative stress. Curr Pharm Des. 2020;26:4246–60. 32640953 10.2174/1381612826666200708133912

[102] Abramov AY Scorziello A Duchen MR. Three distinct mechanisms generate oxygen free radicals in neurons and contribute to cell death during anoxia and reoxygenation. J Neurosci. 2007;27:1129–38. 17267568 10.1523/JNEUROSCI.4468-06.2007 PMC6673180

[103] Peripheral arterial disease in people with diabetes. Diabetes Care. 2003;26:3333–41. 14633825 10.2337/diacare.26.12.3333

[104] Hirsch AT Criqui MH Treat-Jacobson D. Peripheral arterial disease detection, awareness, and treatment in primary care. JAMA. 2001;286:1317–24. 11560536 10.1001/jama.286.11.1317

[105] Reaven PD Sacks J. Coronary artery and abdominal aortic calcification are associated with cardiovascular disease in type 2 diabetes. Diabetologia. 2005;48:379–85. 15688207 10.1007/s00125-004-1640-z

[106] Walsh JA Ning H Liu K. Prevalence of electrocardiographic abnormalities in a middle-aged, biracial population: coronary artery risk development in young adults study. J Electrocardiol. 2010;43:385.e1–9. 10.1016/j.jelectrocard.2010.02.001 PMC3569004 20374967

[107] Thiruvoipati T Kielhorn CE Armstrong EJ. Peripheral artery disease in patients with diabetes: epidemiology, mechanisms, and outcomes. World J Diabetes. 2015;6:961–9. 26185603 10.4239/wjd.v6.i7.961 PMC4499529

[108] Lange S Diehm C Darius H. High prevalence of peripheral arterial disease but low antiplatelet treatment rates in elderly primary care patients with diabetes. Diabetes Care. 2003;26:3357–8. 14633834 10.2337/diacare.26.12.3357

[109] Diehm C Schuster A Allenberg JR. High prevalence of peripheral arterial disease and co-morbidity in 6880 primary care patients: cross-sectional study. Atherosclerosis. 2004;172:95–105. 14709362 10.1016/s0021-9150(03)00204-1

[110] Hiatt WR Fowkes FG Heizer G. Ticagrelor versus clopidogrel in symptomatic peripheral artery disease. N Engl J Med. 2017;376:32–40. 27959717 10.1056/NEJMoa1611688

[111] Weragoda J Seneviratne R Weerasinghe MC. Risk factors of peripheral arterial disease: a case control study in Sri Lanka. BMC Res Notes. 2016;9:508. 27938397 10.1186/s13104-016-2314-x PMC5148875

[112] Majid Khan A Lohana P Anvekar P. Risk factors of peripheral vascular disease in diabetes mellitus in Abbottabad, Pakistan: a cross-sectional study. Cureus. 2021;13:e17556. 34646613 10.7759/cureus.17556 PMC8480069

[113] Paneni F Beckman JA Creager MA. Diabetes and vascular disease: pathophysiology, clinical consequences, and medical therapy: part I. Eur Heart J. 2013;34:2436–43. 23641007 10.1093/eurheartj/eht149 PMC3743069

[114] Ferris FL 3rd. How effective are treatments for diabetic retinopathy? JAMA. 1993;269:1290–1. 8437309

[115] Harris MI Klein R Welborn TA. Onset of NIDDM occurs at least 4-7 yr before clinical diagnosis. Diabetes Care. 1992;15:815–9. 1516497 10.2337/diacare.15.7.815

[116] Porta M Curletto G Cipullo D. Estimating the delay between onset and diagnosis of type 2 diabetes from the time course of retinopathy prevalence. Diabetes Care. 2014;37:1668–74. 24705614 10.2337/dc13-2101

[117] Kianpour F Fararouei M Hassanzadeh J. Performance of diabetes screening tests: an evaluation study of Iranian diabetes screening program. Diabetol Metab Syndr. 2021;13:13. 33499908 10.1186/s13098-021-00632-9 PMC7836149

[118] Mathur D Santoyo-Olsson J Stewart A. Using capillary blood glucose for eligibility screening in community-based diabetes prevention study. Paper presented at: Diabetes. 2008.

[119] Nair ATN Wesolowska-Andersen A Brorsson C. Heterogeneity in phenotype, disease progression and drug response in type 2 diabetes. Nat Med. 2022;28:982–8. 35534565 10.1038/s41591-022-01790-7

[120] Li L Cheng WY Glicksberg BS. Identification of type 2 diabetes subgroups through topological analysis of patient similarity. Sci Transl Med. 2015;7:311ra174. 10.1126/scitranslmed.aaa9364 PMC4780757 26511511

[121] Ahlqvist E Storm P Käräjämäki A. Novel subgroups of adult-onset diabetes and their association with outcomes: a data-driven cluster analysis of six variables. Lancet Diabetes Endocrinol. 2018;6:361–9. 29503172 10.1016/S2213-8587(18)30051-2

[122] Meng Y Li S Khan J. Sugar- and artificially sweetened beverages consumption linked to type 2 diabetes, cardiovascular diseases, and all-cause mortality: a systematic review and dose-response meta-analysis of prospective cohort studies. Nutrients. 2021;13:2636. 34444794 10.3390/nu13082636 PMC8402166

[123] Nylen ES Kokkinos P Myers J. Prognostic effect of exercise capacity on mortality in older adults with diabetes mellitus. J Am Geriatr Soc. 2010;58:1850–4. 20929462 10.1111/j.1532-5415.2010.03068.x

[124] Hsu CC Hwang SJ Tai TY. Cigarette smoking and proteinuria in Taiwanese men with Type 2 diabetes mellitus. Diabet Med. 2010;27:295–302. 20536492 10.1111/j.1464-5491.2010.02947.x

[125] Martín-Timón I Sevillano-Collantes C Segura-Galindo A. Type 2 diabetes and cardiovascular disease: have all risk factors the same strength? World J Diabetes. 2014;5:444–70. 25126392 10.4239/wjd.v5.i4.444 PMC4127581

[126] Śliwińska-Mossoń M Milnerowicz H. The impact of smoking on the development of diabetes and its complications. Diabetes Vasc Dis Res. 2017;14:265–76. 10.1177/1479164117701876 28393534

[127] Sung YT Hsiao CT Chang IJ. Smoking cessation carries a short-term rising risk for newly diagnosed diabetes mellitus independently of weight gain: a 6-year retrospective cohort study. J Diabetes Res. 2016;2016:3961756. 27478846 10.1155/2016/3961756 PMC4960337

[128] Selvin E Marinopoulos S Berkenblit G. Meta-analysis: glycosylated hemoglobin and cardiovascular disease in diabetes mellitus. Ann Intern Med. 2004;141:421–31. 15381515 10.7326/0003-4819-141-6-200409210-00007

[129] Sarwar N Aspelund T Eiriksdottir G. Markers of dysglycaemia and risk of coronary heart disease in people without diabetes: reykjavik prospective study and systematic review. PLoS Med. 2010;7:e1000278. 20520805 10.1371/journal.pmed.1000278 PMC2876150

[130] Gæde P Vedel P Larsen N. Multifactorial intervention and cardiovascular disease in patients with type 2 diabetes. N Engl J Med. 2003;348:383–93. 12556541 10.1056/NEJMoa021778

[131] Ismail-Beigi F Craven T Banerji MA. Effect of intensive treatment of hyperglycaemia on microvascular outcomes in type 2 diabetes: an analysis of the ACCORD randomised trial. Lancet (London, England). 2010;376:419–30. 20594588 10.1016/S0140-6736(10)60576-4 PMC4123233

[132] Duckworth W Abraira C Moritz T. Glucose control and vascular complications in veterans with type 2 diabetes. N Engl J Med. 2009;360:129–39. 19092145 10.1056/NEJMoa0808431

[133] Kearney PM Blackwell L Collins R. Efficacy of cholesterol-lowering therapy in 18,686 people with diabetes in 14 randomised trials of statins: a meta-analysis. Lancet (London, England). 2008;371:117–25. 18191683 10.1016/S0140-6736(08)60104-X

[134] Markovic TP Jenkins AB Campbell LV. The determinants of glycemic responses to diet restriction and weight loss in obesity and NIDDM. Diabetes Care. 1998;21:687–94. 9589225 10.2337/diacare.21.5.687

[135] Patel A MacMahon S Chalmers J. Intensive blood glucose control and vascular outcomes in patients with type 2 diabetes. N Engl J Med. 2008;358:2560–72. 18539916 10.1056/NEJMoa0802987

[136] Gerstein HC Miller ME Byington RP. Effects of intensive glucose lowering in type 2 diabetes. N Engl J Med. 2008;358:2545–59. 18539917 10.1056/NEJMoa0802743 PMC4551392

[137] Tandon N Ali MK Narayan KM. Pharmacologic prevention of microvascular and macrovascular complications in diabetes mellitus: implications of the results of recent clinical trials in type 2 diabetes. Am J Cardiovasc Drugs. 2012;12:7–22. 22217193 10.2165/11594650-000000000-00000

[138] Marshall SM Flyvbjerg A. Prevention and early detection of vascular complications of diabetes. BMJ. 2006;333:475–80. 16946335 10.1136/bmj.38922.650521.80 PMC1557968

[139] Khan T Bertram MY Jina R. Preventing diabetes blindness: cost effectiveness of a screening programme using digital non-mydriatic fundus photography for diabetic retinopathy in a primary health care setting in South Africa. Diabetes Res Clin Pract. 2013;101:170–6. 23796361 10.1016/j.diabres.2013.05.006

[140] Wells JA Glassman AR Ayala AR. Aflibercept, bevacizumab, or ranibizumab for diabetic macular edema. N Engl J Med. 2015;372:1193–203. 25692915 10.1056/NEJMoa1414264 PMC4422053

[141] Mogensen CE Christensen CK Vittinghus E. The stages in diabetic renal disease. With emphasis on the stage of incipient diabetic nephropathy. Diabetes. 1983;32(Suppl 2):64–78. 6400670 10.2337/diab.32.2.s64

[142] Molitch ME DeFronzo RA Franz MJ. Nephropathy in diabetes. Diabetes Care. 2004;27:S79–83. 14693934 10.2337/diacare.27.2007.s79

[143] Stephenson JM Fuller JH. Microalbuminuria is not rare before 5 years of IDDM. EURODIAB IDDM Complications Study Group and the WHO Multinational Study of Vascular Disease in Diabetes Study Group. J Diabetes Complications. 1994;8:166–73. 8086653 10.1016/1056-8727(94)90035-3

[144] Caramori ML Fioretto P Mauer M. Low glomerular filtration rate in normoalbuminuric type 1 diabetic patients: an indicator of more advanced glomerular lesions. Diabetes. 2003;52:1036–40. 12663477 10.2337/diabetes.52.4.1036

[145] MacIsaac RJ Tsalamandris C Panagiotopoulos S. Nonalbuminuric renal insufficiency in type 2 diabetes. Diabetes Care. 2004;27:195–200. 14693989 10.2337/diacare.27.1.195

[146] Gaspari F Perico N Remuzzi G. Measurement of glomerular filtration rate. Kidney Int Suppl. 1997;63:S151–4. 9407445

[147] Friedman R De Azevedo MJ Gross JL. Is endogenous creatinine clearance still a reliable index of glomerular filtration rate in diabetic patients? Braz J Med Biol Res. 1988;21:941–4. 3150296

[148] Selby NM Taal MW. An updated overview of diabetic nephropathy: diagnosis, prognosis, treatment goals and latest guidelines. Diabetes Obes Metab. 2020;22(Suppl 1):3–15. 10.1111/dom.14007 32267079

[149] Quiroga B Arroyo D de Arriba G. Present and future in the treatment of diabetic kidney disease. J Diabetes Res. 2015;2015:801348. 25945357 10.1155/2015/801348 PMC4405221

[150] Arora MK Singh UK. Molecular mechanisms in the pathogenesis of diabetic nephropathy: an update. Vascul Pharmacol. 2013;58:259–71. 23313806 10.1016/j.vph.2013.01.001

[151] Tommerdahl KL Shapiro ALB Nehus EJ. Early microvascular complications in type 1 and type 2 diabetes: recent developments and updates. Pediatr Nephrol (Berlin, Germany). 2022;37:79–93. 10.1007/s00467-021-05050-7 PMC8527882 33852054

[152] Jaiswal M Divers J Urbina EM. Cardiovascular autonomic neuropathy in adolescents and young adults with type 1 and type 2 diabetes: the SEARCH for Diabetes in Youth Cohort Study. Pediatr Diabetes. 2018;19:680–9. 29292558 10.1111/pedi.12633 PMC5938122

[153] Rendell MS. The time to develop treatments for diabetic neuropathy. Expert Opin Investig Drugs. 2021;30:119–30. 10.1080/13543784.2021.1868433 33423557

[154] Daniel V Daniel K. Diabetic neuropathy: new perspectives on early diagnosis and treatments. J Curr Diabetes Reports. 2022;3:12–4.

